# The War on Rats

**Published:** 1908-07-11

**Authors:** A. E. Moore

**Affiliations:** Secretary. The Society for the Destruction of Vermin. 95 Wigmore Street, London, W.


### EDITOR'S LETTER-BOX.

### THE WAR ON RATS.

Sir,—In order to obtain accurate information regarding the nature and extent of the damage done by rats within the United Kingdom, my committee have prepared a schedule of questions which they desire to place into the hands of all those who are in a position, from their own experience, to give valuable information concerning temporary or permanent rat plagues in their districts, the damage inflicted by rats, the steps taken by them—individually or in co-operation with others—for preventing such damage, the means chosen for that purpose, and the results obtained.

As the only means of gathering important knowledge of that kind is by favour of the Press—short of undertaking the appalling task of sending the questions to everyone likely to suffer, or to have suffered, loss through rats—that is, to every householder in the country—my committee venture to hope that you will permit an appeal to your readers to support the Society by asking for a copy of the schedule and returning it with such information as they may be able to impart.

I enclose a copy of the schedule for your information, and I am, Sir, your obedient servant,

A. E. Moore, Secretary.

The Society for the Destruction of Vermin.
95 Wigmore Street, London, W., July 7, 1908.

